# Diverse molecular signatures for ribosomally ‘active’ Perkinsea in marine sediments

**DOI:** 10.1186/1471-2180-14-110

**Published:** 2014-04-29

**Authors:** Aurélie Chambouvet, Cédric Berney, Sarah Romac, Stéphane Audic, Finlay Maguire, Colomban De Vargas, Thomas A Richards

**Affiliations:** 1 Life Sciences, The Natural History Museum, Cromwell Road, London SW7 5BD, UK; 2 CNRS, UMR7144, Equipe Evolution du Plancton et Paleo-Ocean, Roscoff, France; 3 UPMC Univ Paris 06, UMR 7144 Adaptation et Diversité en Milieu Marin, Roscoff, France; 4 Biosciences, University of Exeter, Geoffrey Pope Building, Exeter EX4 4QD, UK

**Keywords:** 454 pyrosequencing, *Perkinsus*, *Parvilucifera*, Food web, Protist, Parasite

## Abstract

**Background:**

Perkinsea are a parasitic lineage within the eukaryotic superphylum Alveolata. Recent studies making use of environmental small sub-unit ribosomal RNA gene (SSU rDNA) sequencing methodologies have detected a significant diversity and abundance of Perkinsea-like phylotypes in freshwater environments. In contrast only a few Perkinsea environmental sequences have been retrieved from marine samples and only two groups of Perkinsea have been cultured and morphologically described and these are parasites of marine molluscs or marine protists. These two marine groups form separate and distantly related phylogenetic clusters, composed of closely related lineages on SSU rDNA trees. Here, we test the hypothesis that Perkinsea are a hitherto under-sampled group in marine environments. Using 454 diversity ‘tag’ sequencing we investigate the diversity and distribution of these protists in marine sediments and water column samples taken from the Deep Chlorophyll Maximum (DCM) and sub-surface using both DNA and RNA as the source template and sampling four European offshore locations.

**Results:**

We detected the presence of 265 sequences branching with known Perkinsea, the majority of them recovered from marine sediments. Moreover, 27% of these sequences were sampled from RNA derived cDNA libraries. Phylogenetic analyses classify a large proportion of these sequences into 38 cluster groups (including 30 novel marine cluster groups), which share less than 97% sequence similarity suggesting this diversity encompasses a range of biologically and ecologically distinct organisms.

**Conclusions:**

These results demonstrate that the Perkinsea lineage is considerably more diverse than previously detected in marine environments. This wide diversity of Perkinsea-like protists is largely retrieved in marine sediment with a significant proportion detected in RNA derived libraries suggesting this diversity represents ribosomally ‘active’ and intact cells. Given the phylogenetic range of hosts infected by known Perkinsea parasites, these data suggest that Perkinsea either play a significant but hitherto unrecognized role as parasites in marine sediments and/or members of this group are present in the marine sediment possibly as part of the ‘seed bank’ microbial community.

## Background

Environmental DNA (eDNA) analyses have demonstrated that diversity records are missing significant data regarding protists (reviewed in: [1-3]). Parasitic protists are a key component of food webs, yet the role and diversity of these groups is often unknown (e.g. [4-6]). The protist ‘superphylum’ Alveolata includes numerous polyphyletic groups of parasites [7], for example: Apicomplexa, Perkinsea (also named perkinsids or Perkinsozoa) and Syndiniales (including both marine alveolate group I and II [also sometimes called MALVI & MALVII a]) [4,8].

Molecular surveys have shown that Perkinsea-like sequences can be diverse and abundant in freshwater lakes, suggesting this group plays an important role in freshwater food webs [5,9-11]. However, most freshwater Perkinsea have still not been characterised ecologically or morphologically, with one exception, a recently identified Perkinsea-like protist linked to local mortality events of the Southern Leopard frog *Rana sphenocephala* in the USA in 2003 [12]. Analysis of the SSU rDNA sequence of this protist suggests that this infectious agent branches close to the Perkinsea in SSU rDNA phylogenies within a cluster consisting of only freshwater environmental sequences [12-14]. With the exception of the Perkinsea associated with frog infections, all morphological descriptions and cultured representatives of the Perkinsea are derived from two marine genera: *Perkinsus* and *Parvilucifera*.

*Perkinsus* is a group of parasites infecting molluscs and includes *P. marinus*, the main cause of mortality of bivalves leading to the economically important shellfish disease ‘Dermo’ [15]. *Parvilucifera* spp. are known to infect up to 26 different dinoflagellates, playing a role in species succession, for example, infecting dinoflagellates that cause red-tides [16]. Taken together these data suggest that the Perkinsea phylum is a diverse group of parasites infecting a wide range of species such as: molluscs, amphibians and dinoflagellates [14].

Numerous clone library surveys of eukaryotic diversity in marine waters have now been published (e.g. [17-22]) yet only a few Perkinsea sequences have been identified. Specifically, only nine sequences belonging to Perkinsea that are distinct from either Perkinsus or Parvilucifera cluster groups are currently available in GenBank (May-2013). To our knowledge, most of the environmental surveys of marine environments have, however, focused on sub-surface or deep chlorophyll maximum (DCM) water column samples, with only a few studies sampling sediments (e.g. [18,23,24]). As such, marine sediments are often thought of as a ‘black box’ in terms of microbial diversity and function [25], lacking in eukaryotic-specific molecular surveys (e.g. [18,23]). Furthermore, the majority of these publications use clone library survey methods and therefore give only a partial view of biodiversity. In contrast second-generation sequencing of environmental sequence tags theoretically allow deeper surveys of microbial biodiversity allowing the detection of low abundance microbes [26,27]. In this paper we use second generation sequencing methods to evaluate the diversity of the Perkinsea in multiple marine environments and test the hypothesis that the Perkinsea are hitherto under sampled group in marine environments.

## Results and discussion

### Processing of sequence data

**Figure 1:**
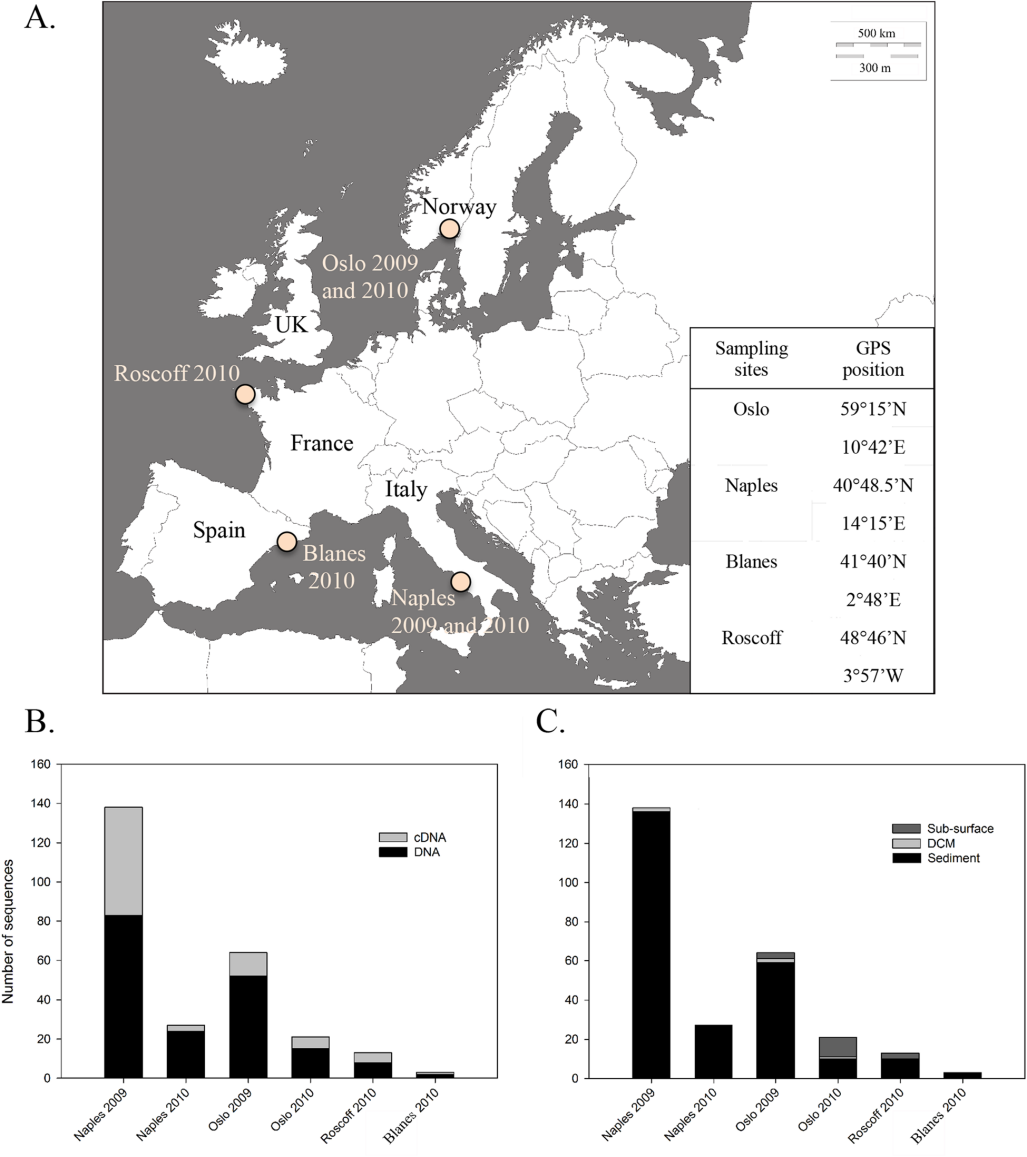
**BioMarKs sampling locations and recovery of Perkinsea-like sequences. A**: Map of BioMarKs sampling locations across Europe with GPS position of the sampling sites. Number of 454 sequences recovered from each sampling site, **B**: Perkinsea sequences recovered from source template (i.e. DNA or cDNA), **C**: Sampling provenance of Perkinsea sequences across sampling location (water column sub-surface, water column DCM or sediment).

Using 454 pyro-sequencing, we investigated the diversity of Perkinsea in a selection of European marine samples, sequencing the V4 region of the SSU rDNA [27] using both rDNA and rRNA as template. A similar DNA-based approach has been used to investigate freshwater Perkinsea [14]. We obtained sequence data from samples collected in four European coastal sites (Figure 1A), including sediment and multiple size filtrates from the sub-surface and the DCM water column samples (using a plankton net for the 2000-20 μm fraction and sequential filtration for 3–20 μm and 0.8-3 μm fractions). All V4 sequence reads (~380 bp) were assigned to eukaryotic taxonomic groups using a custom-built pipeline developed by the BioMarKs consortium (see [28]). This analysis identified 271 sequences preliminarily classified as Perkinsea.

The taxonomic affiliation of these sequences was checked using phylogenetic analyses of a SSU rDNA dataset comprising representative alveolate groups (Additional file 1: Figure S1 and Additional file 2: Table S1) and environmental sequences retrieved from GenBank (Additional file 3: Table S2). Of the 271 sequences initially identified as Perkinsea, alignment based analyses and phylogeny confirmed that 265 individual 454 ‘tag’ sequences were not chimeras [29] and branched with known Perkinsea sequences. Moreover, a large number of the 265 sequences were highly similar and so were clustered at 99% identity resulting in 150 unique V4 sequences branching within, or close to, Perkinsea taxonomic groups (Additional file 1: Figure S1). Table 1 summarises the provenance of the sequences sampled and provides information regarding the total % of Perkinsea sequences within each V4 sequence dataset, which ranges from 0.244% to 0.006% of the 454 sequencing effort from each of the environments sampled.

**Table 1:** Summary of samples used for 454 sequencing

**Geographic site**	**Year**	**Depth**	**Size fraction**	**DNA**	**RNA**
Naples	2009	Sediment	Total	84 (0.243%)	54 (0.264%)
		DCM^a^	0.8-3 μm	2 (0.008%)	0
		Sub-surface	-	0	0
Naples	2010	Sediment	Total	24 (0.141%)	3 (0.043%)
		DCM^a^	-	0	0
		Sub-surface	-	0	0
Oslo	2009	Sediment	Total	50 (0.145%)	7 (0.024%)
		DCM^a^	0.8-3 μm	1 (0.006%)	0
			3-20 μm	1(0.007%)	0
		Sub-surface	0.8-3 μm	3 (0.032%)	0
Oslo	2010	Sediment	Total	9 (0.056%)	1 (0.008%)
		DCM^a^	0.8-3 μm	1 (0.007%)	0
		Sub-surface	0.8-3 μm	3 (0.019%)	0
			20 μm-total	3 (0.021%)	4 (0.034%)
Barcelona	2010	Sediment	Total	1 (0.105%)	1 (0.023%)
		DCM^a^	-	0	0
		Sub-surface	-	0	0
Roscoff	2010	Sediment	Total	8 (0.112%)	2 (0.069%)
		DCM^a^	-	0	0
		Sub-surface	0.8-3 μm	3 (0.027%)	0

The table includes the number of Perkinsea sequences recovered while the figures given in the brackets correspond to the percentage of Perkinsea sequences compared to the total sequencing effort for each sample.

^a^Deep chlorophyll maximum.

### Diversity within marine Perkinsea

**Figure 2:**
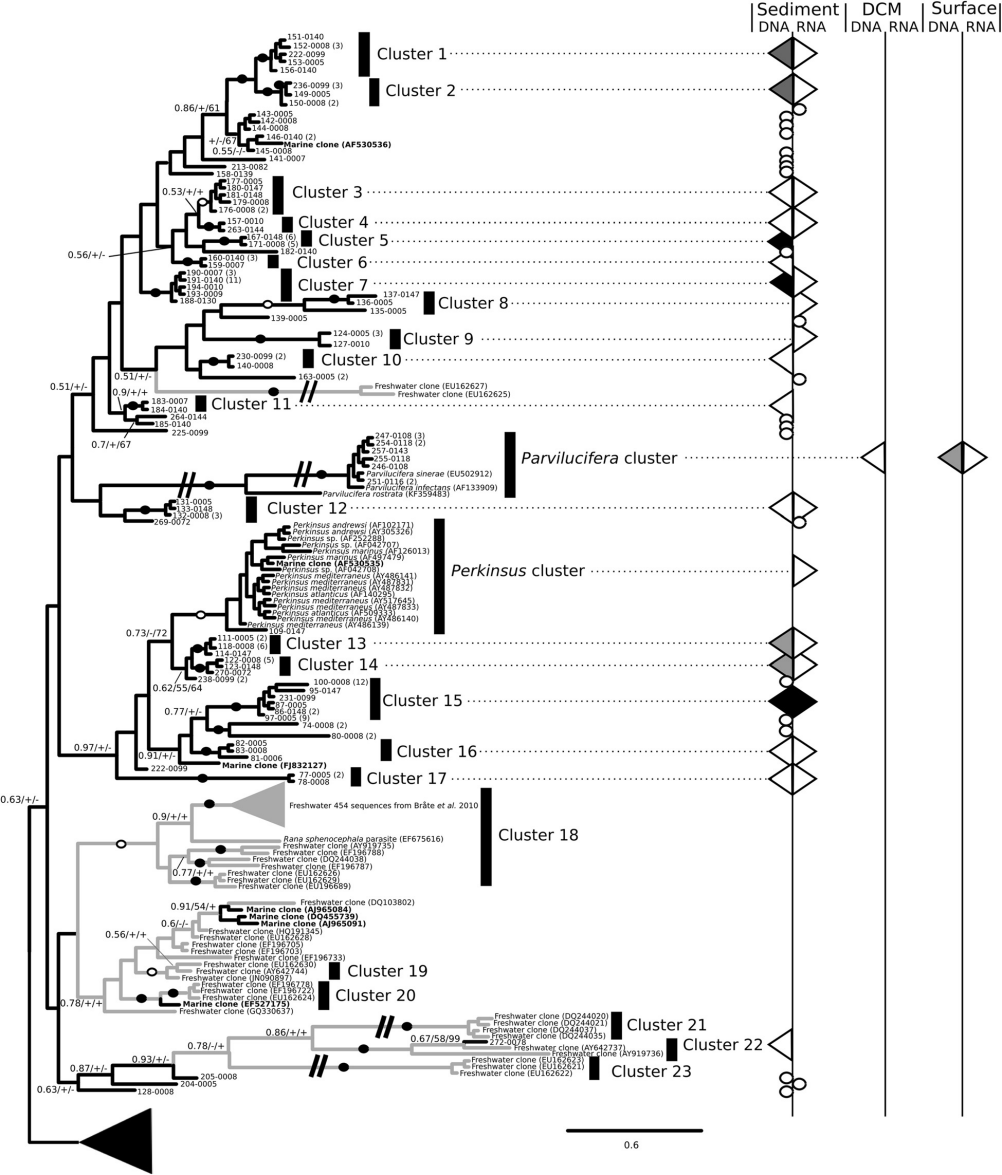
**Bayesian phylogenetic tree of Perkinsea diversity using the V4 region of SSU rDNA gene (part 1) and provenance of the Perkinsea BioMarKs sequences.** Subsection of the phylogenetic tree is shown (see Figure 3 for the rest of the phylogeny). Bayesian posterior probability, Maximum Likelihood bootstrap (1,000 replicates), and LogDet distance bootstrap (1,000 replicates) values are added at each node using the following convention: support values are summarised by black circles on the branch when support are equal to or higher than 0.9/80%/80% and ringed circles when bootstrap values are between 0.6/60%/60% and 0.9/80%/80%. When the bootstrap value is below 60%, a “+” is added if the topology of the tree is recovered in the ML and LogDet analyses. A “-” is shown when these tree topologies are not consistent. Nine sequences of Dinoflagellata and five sequences of Marine Alveolata group II were used as an outgroup. Branches shortened by ½ are labelled with a double slashed line. The black and grey branches on the tree indicate marine and freshwater lineages respectively. Distribution and provenance of sequences across RNA and DNA derived libraries are illustrated down the right columns as shaded triangles if they represent a cluster group. Number in brackets refers to multiple identical sequence reads from the same sample. Circles are used to represent the provenance of a single environment unique sequence cluster. The colour of triangles designates the number of sequences recovered from each location (surface, DCM and sediment) or rDNA/rRNA for each cluster group. White represent between 0 and 5 sequences, Grey between 6–10 and black higher than 10. For correspondence between the 17 freshwater cluster groups identified by Bråte *et al. *(2010) and the 5 freshwater cluster groups identified in our analyses see Additional file 4: Table S3.

**Figure 3:**
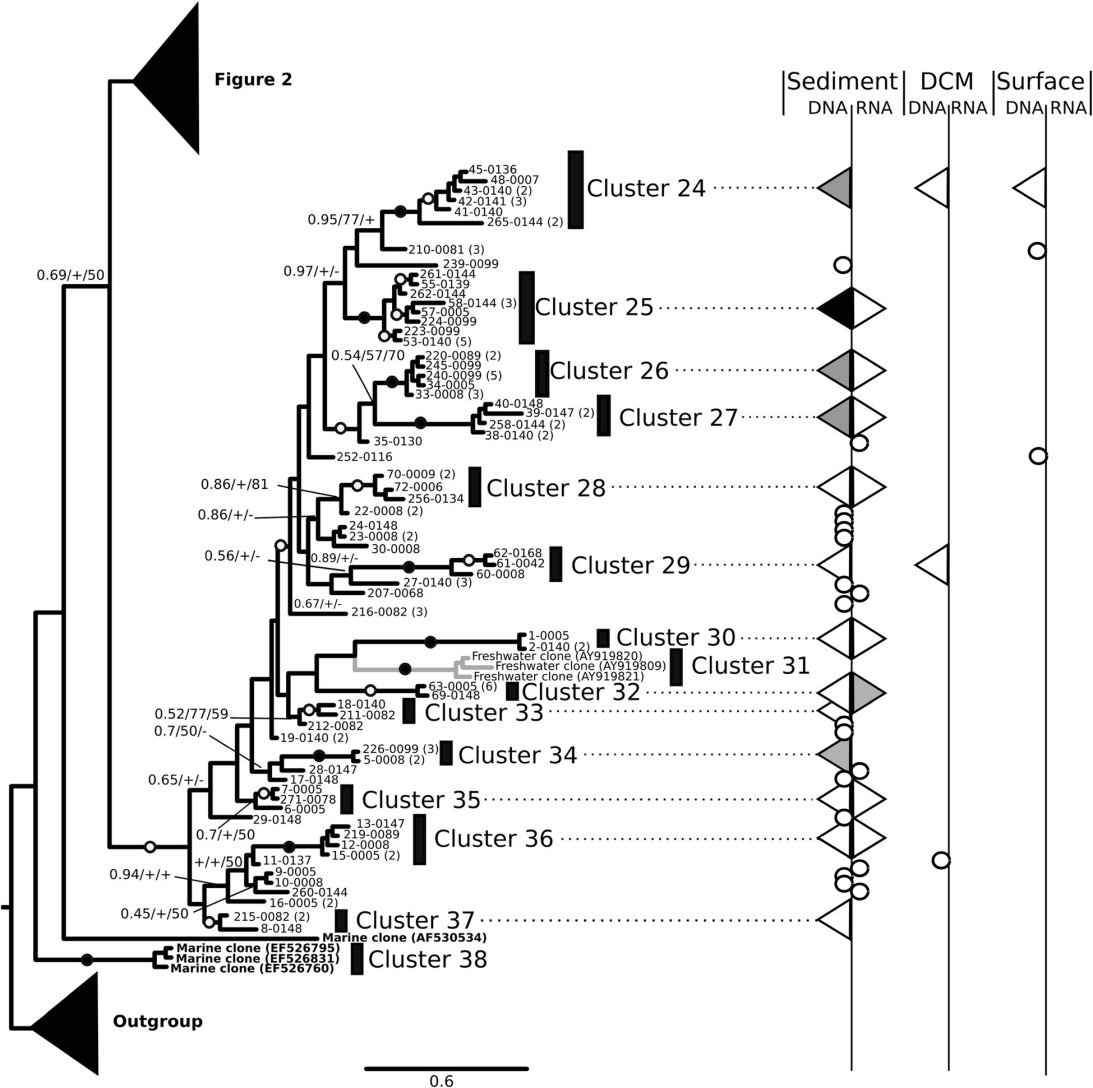
**Bayesian phylogenetic tree of Perkinsea diversity using the V4 region of SSU rDNA gene (part 2) and provenance of the Perkinsea BioMarKs sequences.** Remaining part of Bayesian phylogenetic tree of Perkinsea diversity using the V4 region of SSU rDNA gene. This phylogeny is illustrated using the same conventions as Figure 2.

To investigate the diversity and environmental distribution of the Perkinsea-like sequence tags, we conducted a phylogenetic analysis focusing on the V4 region and including the 150 sequence clusters identified (Figures 2 and 3). The regions flanking the variable V4 region are relatively conserved, while V4 stems and loops are variable [27,30]. The phylogeny was derived from a masked alignment of 330 characters and included a mixture of sites with fast and slow patterns of variation. As our analysis was limited to the V4 region the deep and intermediate nodes of the phylogeny are poorly resolved so that the tree is only helpful for demonstrating the diversity of Perkinsea-like sequences and not the internal topology of the Perkinsea group, consistent with the aim of this study.

To identify a conservative picture of Perkinsea diversity we classified the sequence diversity into ‘cluster-groups’ on the basis of two restrictive criteria: 1) moderate topology support (>0.6/60%/60%) and 2) possession of two sequences from separate samples. Using this approach we identified 38 phylogenetic clusters labeled as cluster 1-38 on Figures 2 and 3 in addition to the morphologically characterised *Perkinsus* and *Parvilucifera* groups. 30 of these clusters represent previously undescribed marine diversity-groups. Additionally, 42 unique sequence clusters (28% - labeled with circles on the right column in Figures 2 and 3) were not grouped into ‘cluster-groups’ using our classification criteria.

The 30 new marine cluster groups show no more than 97% sequence identity between each group, suggesting they constitute taxonomically distinct groups. In contrast, the four described species that belong to *Perkinsus* spp. have highly similar SSU rDNA sequences (>98%, [31]). If the pattern of SSU rDNA variation in *Perkinsus* species is consistent across the wider diversity detected here, then these 30 cluster groups are likely to represent a diversity of forms with distinct biological and/or ecological traits, i.e. given the biology of the known Perkinsea species the diversity of sequences detected here putatively represent parasites infecting a range of host organisms.

### Marine freshwater transitions

A growing body of literature has addressed the frequency in which protist groups have spread between marine and freshwater environments (e.g. [32,33]) with varying perspectives on the number and relative ‘ease’ of these transitions dependant on the group studied and the criteria used for identifying these transitions [14,32]. As described by Bråte *et al.* in 2010, marine-freshwater transitions are likely to have occurred during the diversification of the Perkinsea [14]. Our phylogenetic analyses identified the distribution of sequences recovered from both marine and freshwater environments demonstrating eight putative transitions on the phylogeny (five into freshwater and 3 into marine environments - Figures 2 and 3). However, we note that only three of these transitions are resolved in our V4 phylogenies with bootstrap support in excess of 50%. As such, additional sequencing from a range of environments combined with robust multi-gene phylogenetic analyses is required to characterise the frequency of freshwater-marine environmental transitions within the Perkinsea.

### The majority of marine Perkinsea diversity is recovered from sediments

244 (92%) of the V4 sequences classified as Perkinsea were recovered from sediment. Moreover, 27% of the total sequencing effort were sampled from RNA derived cDNA libraries (Figure 1B,C and Table 1) suggesting that a significant proportion of the Perkinsea sequences were recovered from ribosomally active and intact cells. A large proportion of published environmental sequences are derived from DNA, a method that potentially detects dead organisms or extracellular DNA [34]. This is an issue arising from eDNA sequence surveys of sediment/soil environments. In contrast, extracellular RNA is thought to be less stable so that the use of rRNA can be useful for identifying ribosomally active microbes, inferring intact cells, but not distinguishing between active, senescent, dormant or encysted cells [35]. Therefore, we cannot exclude the possibility that the Perkinsea detected here are in a dormancy period or ‘dying’ whilst still maintaining transcription of a detectable RNA profile. However, these analyses identify a diverse range of ribosomally active Perkinsea in marine sediments, while in contrast recovering very little evidence of Perkinsea in the water column.

## Conclusions

It has previously been suggested that Syndiniales, including Marine Alveolata group I and II, predominate as parasitic protists in marine waters [36] while it has been suggested that in freshwater environments Perkinsea might play analogous roles in terms of diversity and abundance [5,8,14]. The data presented here indicate that the situation is not so clear-cut and supports the conclusion that Perkinsea are a hitherto under-sampled, diverse, widespread and active group in marine sediments, although the abundance and diversity appears to be somewhat lower than that observed for Syndiniales in marine water column samples [4,8,10,36]. In contrast, the Perkinsea, apart from previously described groups, appear largely absent in the four European marine water columns sampled here. Although we note that absence in the water column may be an artefact produced by: 1) limited detection of Perkinsea by the sequencing methods employed here which is likely to be abundance-dependant and therefore prone to miss low abundance groups, and 2) an incomplete sampling of the environments, for example exclusion of certain size fractions, time series, and sampling across a diversity of abiotic gradients.

These results are based on 454 methods targeting a broad spectrum of eukaryotes followed by bioinformatics extraction of Perkinsea-like sequences. Such approaches can lead to partial detection of target groups, dependant on level of sequence saturation achieved and comprehensiveness of the primers selected. In reality achieving single gene-marker primers that allow comprehensive sampling combined with sample saturation is experimentally difficult, unless a narrow group is targeted. As such, future work should incorporate a multiple -group specific- primer approach in order to improve sampling of Perkinsea diversity and map their environmental distribution. A major challenge of future work is to elucidate the ecological roles of this diversity of Perkinsea putative parasites revealed by eDNA surveys.

## Methods

### Sampling

Four European coastal stations were sampled (Figure 1A) as part of the work of the BioMarKs consortium (http://biomarks.scrol.fr): offshore Oslo (Norway, GPS position 59°15′N, 10°42′E), Naples (Italy, GPS position 40°48.5′N, 14°15′E), Blanes near Barcelona (Spain, GPS position 41°40′N, 2°48′E) and Roscoff (France, GPS position 48°46′N, 3°57′W). Each station was sampled over three depths (sediment, DCM and sub-surface) using the same sampling protocol (as described in [37] - Figure 1A). Environmental conditions and sampling area are described in [28]. Briefly, 30 to 50 litres of seawater were collected at the sub-surface and DCM either using a plankton net (for the fraction between 20–2,000 μm) or using Niskin bottles (for sampling of fractions less than 20 μm) coupled to a CTD sensor. Water samples were then size-fractioned using different pore size polycarbonate filters of 142 nm diameter (20 μm, between 3–20 μm and finally between 0.8-3 μm). Each filter was flash frozen and stored at −80°C for further analysis. Sediment samples were taken from a sediment core. Small aliquots of the surface sediment material (~1 cm^3^) were frozen and stored at −80°C for molecular analysis.

### DNA/RNA extraction and 454 tag sequencing

For water column samples, DNA and RNA were extracted simultaneously using the NucleoSpin RNA L kit (Macherey-Nagel, Düren, Germany). For sediment samples, DNA and RNA were isolated using the PowerMax Soil DNA Isolation kit and the PowerSoil total RNA Isolation kit (MoBio, USA). DNA and RNA quality were confirmed using gel electrophoresis (1.5% agarose gels) and quantified using a NanoDrop ND-1000 Spectrophotometer. To avoid contamination by DNA in the RNA extractions, DNAse from the TurboDNA kit (Ambion, Carlsbad, CA, USA) was used to remove traces of DNA. Extracted RNA (100 ng) was reverse transcribed into cDNA using random primers and the Superscript III RT kit (Invitrogen, Carlsbad, CA, USA) following the protocol outlined by the manufacturer.

Universal eukaryotic primers TAReuk454FWD1 (5′-CCAGCASCYGCGGTAATTCC-3′) and TAReukREV3 (5′-ACTTTCGTTCTTGATYRA-3′) were used to sample the V4 region (~380 bp) of the SSU rDNA [27] using polymerase chain reaction (PCR) amplification. The primers were adapted for 454 sequencing with an A-adapter-tag forward and a B-adapter reverse as outlined in the 454 sequencing instructions. PCRs were performed in 25 μl mixtures of 1X Master Mix fusion High Fidelity DNA polymerase (Finnzymes, Thermo Scientific, Espoo, Finland), 0.35 μM of each primer, 3% dimethyl sulfoxide and 5 ng of template DNA or cDNA. PCR reactions consisted of an initial denaturation step at 98°C for 30s, followed by 10 cycles of: 10s at 98°C, 30s at 53°C and 30s at 72°C and then 15 cycles of 10s at 98°C, 30s at 48°C and 30s at 72°C. All PCR products were conducted in triplicate, checked using agarose gel electrophoresis (1.5% agarose gels), pooled and purified using NucleoSpinExtract II (Macherey-Nagel, Düren, Germany), eluted in 30 μl of water, and quantified using NanoDrop ND-1000 spectrophotometer. A final quantity of 200 ng of PCR product was then selected for 454 sequencing. Amplicon sequencing was carried out using a 454 GS FLX Titanium system (454 life sciences, Branford, USA) installed at Genoscope (http://www.genoscope.cns.fr), France.

### Analysis of 454 reads of the V4 area SSU and phylogenetic analysis

Only reads with exact forward and reverse primer sequences and an estimated sequence error of ≤ 0.1% were retained for further analysis. Reads were assigned to taxonomic groups by co-clustering of sample sequences with those from a custom-built reference SSU rDNA database PR^2^[38] truncated to the V4 region. Reads were assigned to the Perkinsea when they were more similar to a reference Perkinsea sequence than to any other sequence in the PR^2^ database, in terms of global alignment identity. This process identified 271 sequences tentatively classified as Perkinsea (each sequence has been labelled with a sequence number followed by the sequencing ID, see Additional file 5: Table S4 for details).

All existing Perkinsea SSU rDNA sequences (both environmental and from cultured organisms) plus a selection of 31 published sequences that encompass all the other major Alveolata lineages were recovered from the NCBI non-redundant nucleotide database and assembled into a reference dataset of 67 sequences (Additional file 2: Table S1 and Additional file 3: Table S2). The Perkinsea 454 V4 sequences were aligned to the previous reference dataset using Muscle [39], as implemented through the multiple alignment-editing program Seaview [39,40]. The alignment was then improved manually, with particular attention to the V4 region. Ambiguously aligned characters were masked and excluded from the alignment prior to phylogenetic analysis. A preliminary tree was used to identify long-branch or highly novel sequences that could potentially represent chimerical sequences. Candidate chimerical sequences were investigated further by visual inspection of the alignment according to methods described by Berney and co-authors [29]. Of the 271 putative Perkinsea 454 tags identified from the Biomarks dataset, 265 marine V4 454 sequences branched with the Perkinsea and were retained for the final analyses (Table 1 and Additional file 5: Table S4). All 271 sequences are available in the European nucleotide archive (https://www.ebi.ac.uk/ena) under accession numbers PRJEB5698. We have included the six putative chimeras in the submission so these can be checked historically, these six sequences have the reference numbers 37–0005, 54–0139, 134–0005, 138–0140, 206–0147 and 268–0287.

Two datasets were then created, with different alignment masks: 1) a dataset encompassing the complete SSU rDNA sequence alignment and including a wide selection of Alveolata lineages (Alveolata SSU dataset composed of 1,437 positions and 377 sequences) and 2) a second dataset restricted to an alignment mask of the V4 region and focusing only on the Perkinsea phylotypes including sequences from Bråte *et al.*[14] (Perkinsea V4 dataset; 330 positions and 351 sequences). As the 454 sequences only encompassed the V4 region, for the first alignment, all missing positions in the Alveolata SSU dataset were encoded as gaps (consistent with the approach used in [14]). Prior to phylogenetic analyses we used the program Modelgenerator v0.85 [41] to determine the best model parameters for the two datasets. For the Alveolata SSU dataset a general time reversible model was selected with a discrete gamma distribution of the substitution rates (8 categories) and a proportion of invariable sites of 0.14 (GTR + Γ + I; gamma distribution shape parameter of 0.32). For the Perkinsea V4 dataset a GTR + Γ model was selected, with a gamma distribution shape parameter of 0.32 and 8 rate categories.

We then conducted Bayesian analyses using MrBayes v3.2.1 [42]. For both datasets we used the covarion parameter and a Γ rate correction with nst = 6 (equivalent to the GTR substitution model). The chains were run for 5,000,000 generations with two replicate tree searches both with 4 chains with a heat parameter of 2. Trees were sampled every 250 generations. In both analyses the MrBayes runs reached a stationary phase by 500 generation samples, and so the first 500 samples were discarded (as the burnin), and a consensus topology calculated from the remaining trees. For both analyses, the covarion model was compared to the non-covarion via Bayesian model comparison. This should be done using Bayes factors (the ratio of the respective marginal likelihoods for the two models) [43]. Unfortunately, the high dimensionality of parameter space makes the marginal likelihood term computationally intractable to evaluate directly. Therefore, the simplest, if somewhat imperfect, method of estimating the marginal likelihood is that of the modified [44] harmonic mean estimator [45] as implemented in the Trace package v1.4 [46] using 1,000 bootstrap pseudo-replicates. These analyses demonstrate that the use of covarion parameters produced an improved tree search (Additional file 6: Table S5).

For both datasets, support for the tree topology was evaluated by the bootstrap method and using the Bayesian posterior probabilities (PP) from the MrBayes runs [42]. Bootstrap support values (BV) were estimated using RAxML v7.0.3 [47], with 1,000 pseudo-replicates. For the Perkinsea V4 dataset, we also conducted a LogDet distance analysis [48] with 1,000 pseudo-replicates, as implemented in the Seaview [40] tree calculation module. This extra analysis was included to account for the possibility of compositional biases in the sequences [49]. We did not conduct LogDet analysis for the Alveolata SSU dataset because the large number of missing characters resulted in poor bootstrap results (which was not an issue for the likelihood and Bayesian analyses).

## Competing interests

The authors declare that they have no competing interests.

## Authors’ contributions

For the BioMarKs project, SR carried out the molecular work and SA the global bioinformatics analyses. CdV and TAR are PI’s on the BioMarKs project. AC and CB constructed the sequence alignment. AC and TAR analysed the data and wrote the manuscript. All authors read and approved the final manuscript.

## Supplementary Material

Additional file 1: Figure S1 Bayesian phylogeny of Alveolata SSU sequences based on the analysis of 98 sequences of 1470 bp and 265 partial sequences from BioMarks V4 sequencing project (~278 bp in length). Posterior probability values and Maximum Likelihood bootstrap values were added at each node (pp/ML bootstrap support). Support values are summarised by black circles on the branch when they are equal to or higher than 0.90/80% and white circle when bootstrap values are between 0.6/60% and 0.9/80. Three ciliates sequences were used as the outgroup. Taxon names are consistent with Bråte et al. 2010. MA corresponds to Marine Alveolates. Arrow identifies the monophyletic Perkinsea clade. Complex clusters of 454 sequences have been reduced to representative triangles, see Figures 2 and 3 for complete phylogenetic data. Click here for file

Additional file 2: Table S1 Details of published sequences of 18S rDNA used in phylogenetic analysis. Click here for file

Additional file 3: Table S2 Published environmental sequences of 18S rDNA belonging to Perkinsea (Alveolata) used in phylogenetic analysis. Sequences sampled from marine environments are highlighted in grey. Click here for file

Additional file 4: Table S3 Correspondence between newly described clusters from the present study and the previous studies [14]. Click here for file

Additional file 5: Table S4 Description of the V4 sequences ID labeling. Each sequence has been labeled by a unique number was given in Figures 2 and 3 (named X) followed by the sampling ID labeling. Click here for file

Additional file 6: Table S5 Bayesian model comparison for method selection in phylogenetic inference. Click here for file
