# Supplementary figures and images for: Diverse molecular signatures for ribosomally ‘active’ Perkinsea in marine sediments
Source: BMC Microbiol. 2014 Apr 29;14:110. doi: 10.1186/1471-2180-14-110 (PMC4044210; doi:10.1186/1471-2180-14-110)

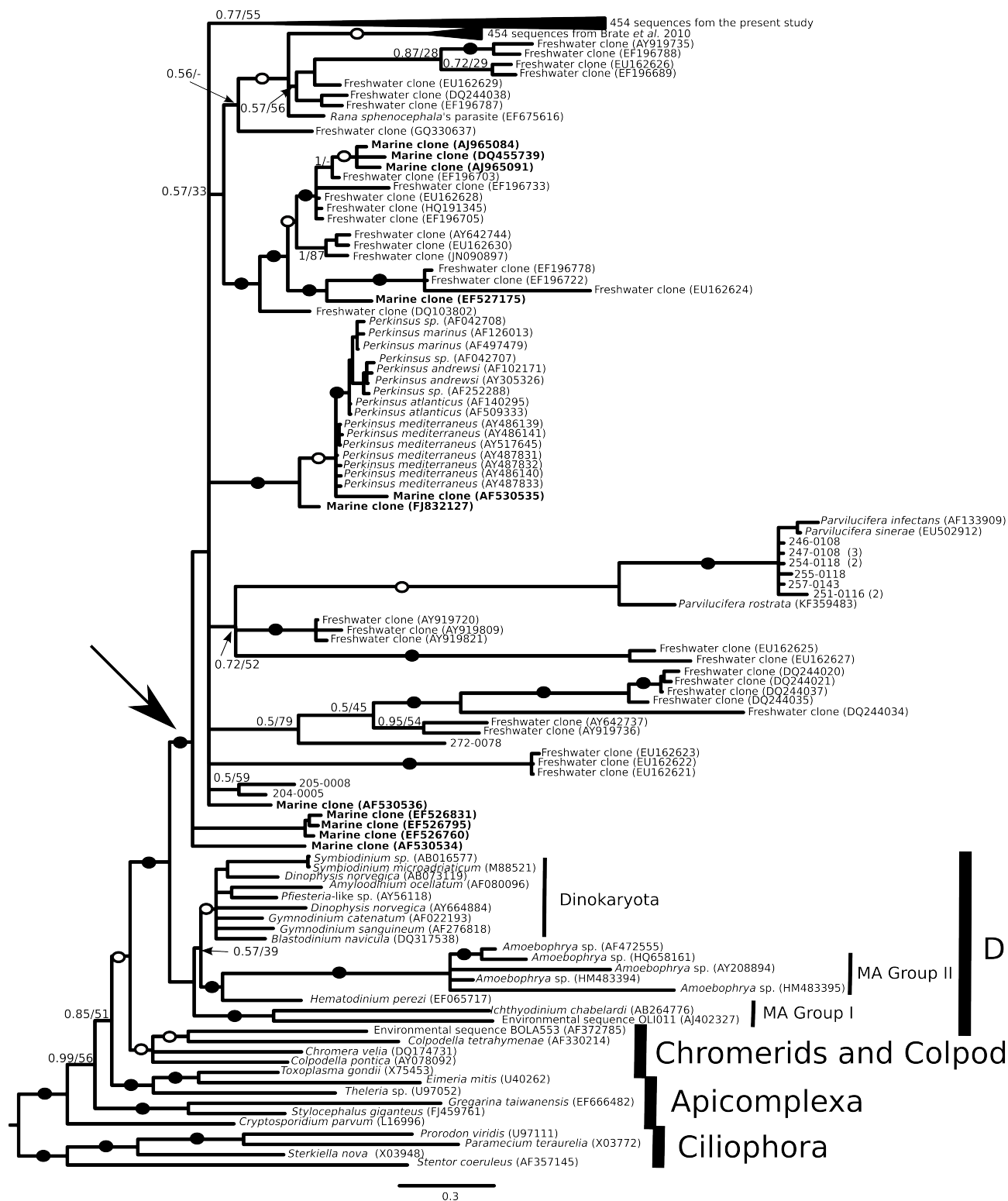

Perkinsea

Supplement: Additional file 1: Figure S1 — Bayesian phylogeny of Alveolata SSU sequences based on the analysis of 98 sequences of 1470 bp and 265 partial sequences from BioMarks V4 sequencing project (~278 bp in length). Posterior probability values and Maximum Likelihood bootstrap values were added at each node (pp/ML bootstrap support). Support values are summarised by black circles on the branch when they are equal to or higher than 0.90/80% and white circle when bootstrap values are between 0.6/60% and 0.9/80. Three ciliates sequences were used as the outgroup. Taxon names are consistent with Bråte et al. 2010. MA corresponds to Marine Alveolates. Arrow identifies the monophyletic Perkinsea clade. Complex clusters of 454 sequences have been reduced to representative triangles, see Figures 2 and 3 for complete phylogenetic data. [file 1471-2180-14-110-S1.pdf]
