# Supplementary material for: Diverse molecular signatures for ribosomally ‘active’ Perkinsea in marine sediments
Source: BMC Microbiol. 2014 Apr 29;14:110. doi: 10.1186/1471-2180-14-110 (PMC4044210; doi:10.1186/1471-2180-14-110)
Supplement: Additional file 2: Table S1 — Details of published sequences of 18S rDNA used in phylogenetic analysis. [file 1471-2180-14-110-S2.docx]

**Supplementary Table 1**: Details of published sequences of 18S rDNA used in phylogenetic analysis.

| **Accession no.** | **Class** | **Order** | **Family/Clade** | **Genus** | **Species** | **Strain/Clone** | **Reference** |
| --- | --- | --- | --- | --- | --- | --- | --- |
| EF675616 | Perkinsea | Perkinsida | * | * | * | *Rana sphenocephala* pathogen | Davis *et al.* 2007 |
| AF126013 | Perkinsea | Perkinsida | * | Perkinsus | marinus | Isolate P1 | Kotob *et al.* 1999 |
| AF497479 | Perkinsea | Perkinsida | * | Perkinsus | marinus | TXsc | Robledo *et al.* 1999 |
| AY486141 | Perkinsea | Perkinsida | * | Perkinsus | mediterraneus | isolate 4 | Casas *et al.* 2004 |
| AF509333 | Perkinsea | Perkinsida | * | Perkinsus | atlanticus | ALG1 | Robledo *et al.* 2002 |
| AF140295 | Perkinsea | Perkinsida | * | Perkinsus | atlanticus | Galicia | Robledo *et al.* 2000 |
| AY486139 | Perkinsea | Perkinsida | * | Perkinsus | mediterraneus | isolate 1 | Casas *et al.* 2004 |
| AY517645 | Perkinsea | Perkinsida | * | Perkinsus | mediterraneus | isolate 9 | Casas *et al.* 2004 |
| AF252288 | Perkinsea | Perkinsida | * | Perkinsus | * | CCA2001 | Coss *et al.* 2001 |
| AF042708 | Perkinsea | Perkinsida | * | Perkinsus | andrewsi | Isolate H49 | Kotob *et al.* 1999 |
| AY305326 | Perkinsea | Perkinsida | * | Perkinsus | andrewsi | ATCC 50807 | Pecher *et al.* 2004 |
| AF042707 | Perkinsea | Perkinsida | * | Perkinsus | sp. | G117 | Kotob *et al.* 1999 |
| AY487832 | Perkinsea | Perkinsida | * | Perkinsus | mediterraneus | Isolate 8 | Casas *et al.* 2004 |
| AY487831 | Perkinsea | Perkinsida | * | Perkinsus | mediterraneus | Isolate 5 | Casas *et al.* 2004 |
| AY487833 | Perkinsea | Perkinsida | * | Perkinsus | mediterraneus | Isolate 12 | Casas *et al.* 2004 |
| AY486140 | Perkinsea | Perkinsida | * | Perkinsus | mediterraneus | Isolate 3 | Casas *et al.* 2004 |
| AF102171 | Perkinsea | Perkinsida | * | Perkinsus | andrewsi |  | Coss *et al.* 2001 |
| AF133909 | Perkinsea | Perkinsida | * | Parvilucifera | infectans |  | Noren *et al.* 1999 |
| EU502912 | Perkinsea | Perkinsida | * | Parvilucifera | sinerae |  | Figueroa *et al.* 2010 |
| KF359483 | Perkinsea | Perkinsida | * | Parvilucifera | rostrata |  | Lepelletier *et al.* 2014 |
| HM483394 | Syndiniales | Amoebophryaceae | * | Amoebophrya | sp. | ex *Gymnodinium instriatum* | Coats *et al.* 2004 |
| HM483395 | Syndiniales | Amoebophryaceae | * | Amoebophrya | sp. | ex *Akashiwo sanguineaum* | Coats *et al.* 2004 |
| HQ658161 | Syndiniales | Amoebophryaceae | * | Amoebophrya | sp. | RCC1626 | Chambouvet *et al.* 2011 |
| AF472555 | Syndiniales | Amoebophryaceae | * | Amoebophrya | sp. | ex *Akashiwo sanguineaum* | Gunderson *et al.* 2002 |
| AY208894 | Syndiniales | Amoebophryaceae | * | Amoebophrya | sp. | ex *Scrippsiella sp.* | John *et al.* unpublished |
| AB016577 | Dinophyceae | Suessiales | Symbiodiniaceae | Symbiodinium | sp. | PLTD-1 | Carlos *et al.* 1999 |
| M88521 | Dinophyceae | Suessiales | Symbiodiniaceae | Symbiodinium | microadriaticum |  | Rowan and Powers 1992 |
| AY456118 | Dinophyceae | Peridiniales | Pfiesteriaceae | Pfiesteria-like | sp. | CCMP1827 | Zhang and Lin 2005 |
| AF022193 | Dinophyceae | Gymnodiniales | Gymnodiniaceae | Gymnodinium | catenatum | MUCC273 | Saunders *et al.* 1997 |
| DQ317538 | Dinophyceae | Blastodiniales | Blastodinium | Blastodinium | navicula |  | Skovgaard *et al.* 2007 |
| AF080096 | Dinophyceae | Blastodiniales | Oodiniaceae | Amyloodinium | ocellatum |  | Litaker *et al.* 1999 |
| AY664884 | Dinophyceae | Dinophysiales | Dinophysiaceae | Dinophysis | norvegica |  | Armbrust *et al.* unpublished |
| AF276818 | Dinophyceae | Gymnodiniales | Gymnodiniaceae | Gymnodinium | sanguineum |  | Gunderson *et al.* 2001 |
| AB264776 | Dinophyceae | Syndiniales | Dino-Group I-Clade 3 | Ichthyodinium | chabelardi |  | Yuasa *et al.* unpublished |
| AJ402327 | Dinophyceae | Syndiniales | Dino-Group I-Clade 5 | * | * | OLI011_75m_11 | Moon-van der Staay *et al.* 2001 |
| EF065717 | Dinophyceae | Syndiniales | Dino-Group IV | Hematodinium | perezi |  | Small et al. 2012 |
| AY664884 | Dinophyceae | Und. | * | * | * | SCM38C60 | Armbrust *et al.* unpublished |
| AF330214 | Apicomplexa | Colpodellidae | * | Colpodella | tetrahymenae |  | Cavalier-Smith *et al.* 2003 |
| AF372785 | Apicomplexa | Colpodellidae | * | * |  | BOLA553 | Dawson and Pace 2002 |
| AY078092 | Apicomplexa | Colpodellidae | * | Colpodella | pontica |  | Kuvardina *et al.* 2002 |
| DQ174731 | Chromerida | * | * | Chromera | velia |  | Moore *et al.* 2008 |
| U40262 | Apicomplexa | Coccidia | Eimeriidae | Eimeria | mitis |  | Relman *et al.* 1996 |
| X75453 | Apicomplexa | Coccidia | Eucoccidiorida | Toxoplasma | gondii |  | Ding *et al.* unpublished |
| U97052 | Apicomplexa | Aconoidasida | Piroplasmida | Theleria | sp. |  | Chae *et al.* 1998 |
| EF666482 | Apicomplexa | Gregarinia | Eugregarinida | Gregarina | taiwanensis |  | Enomoto *et al.* unpublished |
| FJ459761 | Apicomplexa | Gregarinia | Eugregarinida | Stylocephalus | giganteus |  | Clopton 2009 |
| L16996 | Apicomplexa | Cryptosporidium | * | Cryptosporidium | parvum |  | Johnson *et al.* 1993 |
| U97111 | Ciliophora | Intramacronucleata | Prorodontida | Prorodon | viridis |  | Hirt *et al.* unpublished |
| X03772 | Ciliophora | Intramacronucleata | Oligohymenophorea | Paramecium | tetraurelia |  | Sogin and Elwood 1986 |
| X03948 | Ciliophora | Intramacronucleata | Spirotrichea | Sterkiella | nova |  | Elwood *et al.* 1985 |
| AF357145 | Ciliophora | Postciliodesmatophora | Heterotrichea | Stentor | coeruleus |  | Zhu *et al.* unpublished |

**References :**

Carlos AA, Baillie BK, Kawachi M, Maruyama T: **Phylogenetic position of *Symbiodinium* (Dinophyceae) isolates from tridacnids (Bivalvia), cardiids (Bivalvia), a sponge (Porifera), a soft coral (Anthozoa), and a free-living strain**. J Phycol 1999 **35:** 1054-1062.

Casas SM, Grau A, Reece KS, Apakupakul K, Azevedo C, Villalba A: ***Perkinsus mediterraneus* n. sp., a protistan parasite of the European flat oyster *Ostrea edulis* from the Balearic Islands, Mediterranean Sea.** Dis Aquat Org 2004 **58:** 231-244.

Cavalier-Smith T, Chao EE, Oates B: **Molecular phylogeny of apicomonad and oxyrrhid zooflagellates and the evolution of Miozoa Alveolata.** J Parasitol 2003 **89:** 1191-1205.

Chae J, Lee J, Kwon O, Holman PJ, Waghela SD, Wagner GG: **Nucleotide sequence heterogeneity in the small subunit ribosomal RNA gene variable V4 region among and within geographic isolates of *Theileria* from cattle, elk and white-tailed deer.** Vet Parasitol 1998 **75:** 41-52.

Chambouvet A, Alves-de-Souza C, Cueff V, Marie D, Karpov S, Guillou L: **Interplay between the parasite *Amoebophrya* sp. (Alveolata) and the cyst formation of the red tide dinoflagellate *Scrippsiella trochoidea*.** Protist 2011 **162**(4): 637-49.

Clopton RE: **Phylogenetic relationships, evolution, and systematic revision of the septate gregarines Apicomplexa: *Eugregarinorida*: *Septatorina*.** Comp Parasitol 2009 **76:** 167-190.

Coats DW, Kim S, Bachvaroff TR, Handy SM, Delwiche CF: ***Tintinnophagus acutus* n. g., n. sp. Phylum Dinoflagellata, an ectoparasite of the ciliate *Tintinnopsis cylindrica* Daday 1887, and its relationship to *Duboscquodinium collini* Grasse 1952.** J Euk Microbiol 2004 **57:** 468-482.

Coss CA., Robledo JAF, Ruiz GM, Vasta GR: **Description of *Perkinsus andrewsi* n.sp. isolated from the baltic clam *Macoma balthica* by characterization of the ribosomal RNA locus and development of a species-specific PCR-based diagnostic assay.** J Euk Microbiol 2001 **48**: 52-61.

Davis AK, Yabsley MJ, Keel MK, Maerz JC: **Discovery of a novel alveolate pathogen affecting southern leopard frogs in Georgia: description of the disease and host effects.** Ecohealth 2007 **4:** 310-317.

Dawson SC, Pace NR: **Novel kingdom-level eukaryotic diversity in anoxic environments.** Proc. Natl. Acad. Sci. USA 2002 **99:** 8324-8329.

Elwood HJ, Olsen GJ, Sogin ML: **The small-subunit ribosomal RNA gene sequences from the hypotrichous ciliates *Oxytricha nova* and *Stylonychia pustulata*.** Mol Biol Evol 1985 **2:** 399-410.

Figueroa RI, Garcés E, Camp J: **Reproductive plasticity and local adaptation in the host–parasite system formed by the toxic *Alexandrium minutum* and the dinoflagellate parasite *Parvilucifera sinerae*.** Harmful algae 2010 **10:** 56-63.

Gunderson JH, Goss SH, Coats DW: **FISH probes for the detection of the parasitic dinoflagellate *Amoebophrya* sp. infecting the dinoflagellate *Akashiwo sanguinea* in Chesapeake Bay.** J Euk Microbiol 2001 **48:** 670-675.

Gunderson JH, John SA, Boman WC, Coats DW: **Multiple strains of the parasitic dinoflagellate *Amoebophrya* exist in Chesapeake Bay.** J Euk Microbiol 2002 **49:** 469-474.

Johnson DW, Pieniazek NJ, Rose JB: **DNA probe and PCR detection of *Cryptosporidium parvum* compared to immunofluorescence assay**. Water Sci Technol 1993 **27:** 77-84.

Kotob SI, McLaughlin SM, Van Berkum P, Faisal M: **Discrimination between two *Perkinsus* spp. isolated from the softshell clam, *Mya arenaria*, by sequence analysis of two internal transcriped spacers regions and the 5.8S ribosomal RNA gene**. Parasitology 1999 **119:** 363-368.

Kuvardina ON, Leander BS, Aleshin VV, Myl'nikov AP, Keeling PJ, Simdyanov TG: **The phylogeny of colpodellids Alveolata using small subunit rRNA gene sequences suggests they are the free-living sister group to Apicomplexans.** J Euk Microbiol 2002 **49:** 498-504.

Lepelletier F, Karpov SA, Le Panse S, Bigeard E, Skovgaard A, Jeanthon C, Guillou L: ***Parvilucifera rostrata* sp. nov. (Perkinsozoa), a Novel Parasitoid that Infects Planktonic Dinoflagellates.** Protist 2014 **165**:31-49.

Litaker RW, Tester PA, Colorni A, Levy MG, Noga EJ: **The Phylogenetic Relationship of *Pfiesteria piscicida*, *Cryptoperidiniopsoid* sp. *Amyloodinoum ocellatum* and a Pfiesteria-like Dinoflagellate to Other Dinoflagellates and Apicomplexans.** J Phycol 1999 **36:** 1379-1389.

Moon-van der Staay SY, De Wachter R, Vaulot D: **Oceanic 18S rDNA sequences from picoplankton reveal unsuspected eukaryotic diversity.** Nature 2001 **409:** 607-610.

Moore RB, Obornik M, Janouskovec J, Chrudimský T, Vancova M, *et al.*: **A photosynthetic alveolate closely related to apicomplexan parasites.** Nature 2008 **451:** 959-963.

Noren F, Moestrup O, Rehnstam-Holm AS: ***Parvilucifera* *infectans* Noren et Moestrup gen. et sp. nov. Perkinsozoa phylum nov.: a parasitic flagellate capable of killing toxic microalgae.** Eur J Protistol 1999 **35:** 233-254.

Pecher WT, Robledo JA, Vasta GR: **Identification of a second rRNA gene unit in the *Perkinsus andrewsi* genome.** J Euk Microbiol 2004 **51:** 234-245.

Relman DA, Schmidt TM, Gajadhar A, Sogin M, Cross J, Yoder K *et al.*: **Molecular phylogenetic analysis of Cyclospora, the human intestinal pathogen, suggests that it is closely related to *Eimeria* species**. J Infect Dis 1996 **173:** 440-445.

Robledo JA, Wright AC, Marsh AG, Vasta GR: **Nucleotide sequence variability in the nontranscribed spacer of the rRNA locus in the oyster parasite *Perkinsus marinus*.** J Parasitol 1999 **85:** 650-656.

Robledo JA, Coss CA, Vasta GR: **Characterization of the ribosomal RNA locus of Perkinsus atlanticus and development of a polymerase chain reaction-based diagnostic assay.** J Parasitol 2000 **86:** 972-978.

Robledo JA, Nunes PA, Cancela ML, Vasta GR: **Development of an *in vitro* clonal culture and characterization of the rRNA gene cluster of *Perkinsus atlanticus*, a protistan parasite of the clam *Tapes decussatus*.** J Euk Microbiol 2002 **49:** 414-422.

Rowan R, Powers DA: **Ribosomal RNA sequences and the diversity of symbiotic dinoflagellates zooxanthellae.** Proc Natl Acad Sci USA 1992 **89:** 3639-3643.

Saunders GW, Hill DR, Sexton JP, Andersen RA: **Small-subunit ribosomal RNA sequences from selected dinoflagellates: testing classical evolutionary hypotheses with molecular systematic methods**. Edition Springer-Verlag Wein, New York, 1997.

Skovgaard A, Massana R, Saiz E: **Parasitic species of the genus *Blastodinium* Blastodiniphyceae are peridinoid dinoflagellates.** J Phycol 2007 **43:** 553-560.

Small HJ, Shields JD, Reece KS, Bateman K, Stentiford GD: **Morphological and molecular characterization of *Hematodinium perezi* Dinophyceae: Syndiniales, a dinoflagellate parasite of the harbour crab, *Liocarcinus depurator***. J Euk Microbiol 2012 **59:** 54-66.

Sogin ML, Elwood HJ: **Primary structure of the *Paramecium tetraurelia* small-subunit rRNA coding region: phylogenetic relationships within the Ciliophora.** J Mol Evol 1986 **23:** 53-60.

Zhang H, Lin S: **Phylogeny of dinoflagellates based on mitochondrial cytochrome b and nuclear small subunit rDNA sequence comparisons.** J Phycol 2005 **41:** 411-420.
