# Supplementary material for: Diverse molecular signatures for ribosomally ‘active’ Perkinsea in marine sediments
Source: BMC Microbiol. 2014 Apr 29;14:110. doi: 10.1186/1471-2180-14-110 (PMC4044210; doi:10.1186/1471-2180-14-110)
Supplement: Additional file 3: Table S2 — Published environmental sequences of 18S rDNA belonging to Perkinsea (Alveolata) used in phylogenetic analysis. Sequences sampled from marine environments are highlighted in grey. [file 1471-2180-14-110-S3.docx]

**Supplementary Table 2**: Published environmental sequences of 18S rDNA belonging to Perkinsea (Alveolata) used in phylogenetic analysis. Sequences sampled from marine environments are highlighted in grey.

| Accession no. | Environmental clone | Environment | Location | Sampled environment | Reference |
| --- | --- | --- | --- | --- | --- |
| EF196778 | B66 | Lake Bourget | Alps, France | mesotrophic lake | Lepère *et al.* 2008 |
| EF196787 | B91 | Lake Bourget | Alps, France | mesotrophic lake | Lepère *et al.* 2008 |
|  |  |  |  |  |  |
| EF196788 | B95 | Lake Bourget | Alps, France | mesotrophic lake | Lepère *et al.* 2008 |
| EF196689 | BA70 | Lake Bourget | Alps, France | mesotrophic lake | Lepère *et al.* 2008 |
| EF196703 | BA330 | Lake Bourget | Alps, France | mesotrophic lake | Lepère *et al.* 2008 |
| EF196705 | BA372 | Lake Bourget | Alps, France | mesotrophic lake | Lepère *et al.* 2008 |
| EF196733 | B48 | Lake Bourget | Alps, France | mesotrophic lake | Lepère *et al.* 2008 |
| EF196722 | B26 | Lake Bourget | Alps, France | mesotrophic lake | Lepère *et al.* 2008 |
| DQ244038 | PAB5AU2004 | Lake Pavin | Massif Central, France | Oligomesotrophic lake | Lefevre *et al.* 2007 |
| EU162621 | PAA10SP2005 | Lake Pavin | Massif Central, France | Oligomesotrophic lake | Lefèvre *et al.* 2008 |
| EU162622 | PAB8SP2005 | Lake Pavin | Massif Central, France | Oligomesotrophic lake | Lefèvre *et al.* 2008 |
| EU162623 | PAB88SP2005 | Lake Pavin | Massif Central, France | Oligomesotrophic lake | Lefèvre *et al.* 2008 |
| EU162625 | PAG5SP2005 | Lake Pavin | Massif Central, France | Oligomesotrophic lake | Lefèvre *et al.* 2008 |
| EU162626 | PAC11SP2005 | Lake Pavin | Massif Central, France | Oligomesotrophic lake | Lefèvre *et al.* 2008 |
| EU162628 | PAD1SP2005 | Lake Pavin | Massif Central, France | Oligomesotrophic lake | Lefèvre *et al.* 2008 |
| EU162627 | PAD12SP2005 | Lake Pavin | Massif Central, France | Oligomesotrophic lake | Lefèvre *et al.* 2008 |
| EU162629 | PAA8SP2005 | Lake Pavin | Massif Central, France | Oligomesotrophic lake | Lefèvre *et al.* 2008 |
| DQ244020 | PAB11AU2004 | Lake Pavin | Massif Central, France | Oligomesotrophic lake | Lefèvre *et al.* 2007 |
| DQ244037 | PAF7AU2004 | Lake Pavin | Massif Central, France | Oligomesotrophic lake | Lefèvre *et al.* 2007 |
| DQ244021 | PAD10AU2004 | Lake Pavin | Massif Central, France | Oligomesotrophic lake | Lefèvre *et al.* 2007 |
| DQ244035 | PAD7AU2004 | Lake Pavin | Massif Central, France | Oligomesotrophic lake | Lefèvre *et al.* 2007 |
| DQ244034 | PAG2AU2004 | Lake Pavin | Massif Central, France | Oligomesotrophic lake | Lefèvre *et al.* 2007 |
| EF527175 | SA2_2G9 | Framvaren Fjord | South-western Norway | Axonic/sulfidic waters | Behnke *et al.* 2010 |
| EU162624 | PAA9SP2005 | Lake Pavin | Massif Central, France | Oligomesotrophic lake | Lefèvre *et al.* 2008 |
| EU162630 | PAC8SP2005 | Lake Pavin | Massif Central, France | Oligomesotrophic lake | Lefèvre *et al.* 2008 |
| HQ191345 | PA2009C9 | Lake Pavin | Massif Central, France | Oligomesotrophic lake | Monchy *et al*. 2011 |
| EU162628 | PAD1SP2005 | Lake Pavin | Massif Central, France | Oligomesotrophic lake | Lefèvre *et al.* 2008 |
| AY642744 | A31 | Lake Aydat | Massif Central, France | Eutrophic lake | Lefranc *et al.* 2005 |
| AY642737 | A20 | Lake Aydat | Massif Central, France | Eutrophic lake | Lefranc *et al.* 2005 |
| EF526795 | NA2_4B10 | Framvaren Fjord | Norway | Anoxic marine waters | Behnke *et al.* 2010 |
| EF526831 | NA1_3C6 | Framvaren Fjord | Norway | Anoxic marine waters | Behnke *et al.* 2010 |
| EF526760 | NA2_2D10 | Framvaren Fjord | Norway | Anoxic marine waters | Behnke *et al.* 2010 |
| AJ965091 | He000327_72482 | Helgoland marine waters | Germany | Marine waters | Medlin *et al* 2006 |
| AJ965084 | He000327_29 | Helgoland marine waters | Germany | Marine waters | Medlin *et al.* 2006 |
| DQ103802 | M2_18C03 | Mariager Fjord | Danish east coast | Anoxic marine waters | Zuendorf *et al.* 2006 |
| AF530536 | AT4-98 | Hydrothermal sediment | Hydrothermal vents, Mid-Atlantic Ridge | Extreme environment | Lopez-Garcia *et al*. 2003 |
| GQ330637 | PR2_3E_17 | Praz Rodet peat bog | Switzerland | Acidic extreme waters | Lara *et al.* 2011 |
| FJ832127 | BW-dinoclone29 | Ship ballast water | Singapore ballast water | Marine waters | Park unpublished |
| DQ455739 | BB01-172.24w | Estuary | USA: Barnegat Bay, NJ | Marine waters | Lim *et al.* unpublished |
| AY919720 | LG10-12 | Lake George | Adirondack Park North-Eastern New York, USA | Oligotrophic lake | Richards *et al.* 2005 |
| AY919735 | LG15-08 | Lake George | Adirondack Park North-Eastern New York, USA | Oligotrophic lake | Richards *et al.* 2005 |
| AY919809 | LG36-11 | Lake George | Adirondack Park North-Eastern New York, USA | Oligotrophic lake | Richards *et al.* 2005 |
| AY919820 | AY919820 | Lake George | Adirondack Park North-Eastern New York, USA | Oligotrophic lake | Richards *et al.* 2005 |
| AY919821 | LG53-06 | Lake George | Adirondack Park North-Eastern New York, USA | Oligotrophic lake | Richards *et al*. 2005 |
| AY919736 | LG15-10 | Lake George | Adirondack Park North-Eastern New York, USA | Oligotrophic lake | Richards *et al.* 2005 |
| AF530534 | IN242 | Microcolonizers | Hydrothermal vents, Mid-Atlantic Ridge | Extreme environment | Lopez Garcia *et al.* 2003 |
| AF530535 | AT2-6 | Microcolonizers | Hydrothermal vents, Mid-Atlantic Ridge | Extreme environment | Lopez Garcia *et al.* 2003 |
| JN090897 | KRL01E37 | Lake Karla | Greece | Lake | Oikonomou *et al.* 2012 |

**References:**

Behnke A, Barger KJ, Bunge J, Stoeck T: **Spatio-temporal variations in protistan communities along an O/HS gradient in the anoxic Framvaren Fjord (Norway).** FEMS Microbiol Ecol 2010 **72:** 89-102.

Lara E, Mitchell EA, Moreira D, Lopez-Garcia P: **Highly diverse and seasonally dynamic protist community in a pristine peat bog**. Protist 2011 **162:** 14-32.

Lefevre E, Bardot C, Noël C, Carrias J-F, Viscogliosi E, Amblard C *et al.*: **Unveiling fungal zooflagellate as members of freshwater picoeukaryotes: evidence from a molecular diversity study in a deep meromictic lake**. Environ Microbiol 2007 **9:** 61-71.

Lefèvre E, Roussel B, Amblard C, Sime-Ngando T: **The molecular diversity of freshwater picoeukaryotes reveals high occurrence of putative parasitoids in the plankton.** Plos One 2008 **3:** E2324.

Lefranc M, Thenot A, Lepere C, Debroas D: **Genetic diversity of small eukaryotes in lakes differing by thier trophic status.** Appl Environ Microbiol 2005 **71:** 5935-5942.

Lopez-Garcia P, Philippe H, Gail F, Moreira D: **Autochthonous eukaryotic diversity in hydrothermal sediment and experimental microcolonizers at the Mid-Atlantic Ridge**. Proc Natl Acad Sci USA 2003 **100:** 697-702.

Medlin LK, Metfies K, Mehl H, Wiltshire K, Valentin K: **Picoeukaryotic plankton diversity at the Helgoland time series site as assessed by three molecular methods.** Microb Ecol 2006 **52:** 53-71.

Monchy S, Sanciu G, Jobard M, Rasconi S, Gerphagnon M, Chabé M *et al* : **Exploring and quantifying fungal diversity in freshwater lake ecosystems using rDNA cloning/sequencing and SSU tag pyrosequencing.** Environ Microbiol 2011 **13:** 1433-1453.

Oikonomou A, Katsiapi M, Karayanni H, Moustaka-Gouni M, Kormas KA: **Plankton microorganisms coinciding with two consecutive mass fish kills in a newly reconstructed lake.** ScientificWorldJournal 2012 **50135.**

Richards TA, Vepritskiy AA, Gouliamova D, Nierzwicki-Bauer SA: **The molecular diversity of freshwater picoeukaryotes from an oligotrophic lake reveals diverse, distinctive and globally dispersed lineages.** Environ Microbiol 2005 **7:** 1413-1425.

Zuendorf A, Bunge J, Behnke A, Barger KJ, Stoeck T: **Diversity estimates of microeukaryotes below the chemocline of the anoxic Mariager Fjord, Denmark.** FEMS Microbiol Ecol 2006 **58:** 476-491.
