# Supplementary material for: Diverse molecular signatures for ribosomally ‘active’ Perkinsea in marine sediments
Source: BMC Microbiol. 2014 Apr 29;14:110. doi: 10.1186/1471-2180-14-110 (PMC4044210; doi:10.1186/1471-2180-14-110)
Supplement: Additional file 4: Table S3 — Correspondence between newly described clusters from the present study and the previous studies [14]. [file 1471-2180-14-110-S4.docx]

**Supplementary Table 3**: Correspondence between newly described clusters from the present study and the previous studies (Bråte *et al.* 2010).

| **Paper from Bråte *et al.* 2010** | **Present study** | **Sampled environments** |
| --- | --- | --- |
| Perk 01 | Cluster 38 | Marine |
| Perk 03 | Cluster 23 | Freshwater |
| Perk 04 +AY919820 | Cluster 31 | freshwater |
| Perk 07+DQ244020+DQ244021+DQ244035 | Cluster 21 | Freswater |
| Perk 08 | Cluster 22 | Freshwater/marine |
| Perk 09+10+11 | Cluster 18 | Freshwater |
| Perk 13+ 14 +EF162624 | Cluster 20 | Freshwater/marine |
| Perk 16 | Cluster 19 | Freshwater |

**Reference:**

Bråte J, Logares R, Berney C, Ree DK, Klaveness D, Jakobsen KS *et al.*: **Freshwater Perkinsea and marine-freshwater colonizations revealed by pyrosequencing and phylogeny of environmental rDNA**. ISME J 2010 **4:** 1144-1153.
