# Supplementary material for: Diverse molecular signatures for ribosomally ‘active’ Perkinsea in marine sediments
Source: BMC Microbiol. 2014 Apr 29;14:110. doi: 10.1186/1471-2180-14-110 (PMC4044210; doi:10.1186/1471-2180-14-110)
Supplement: Additional file 5: Table S4 — Description of the V4 sequences ID labeling. Each sequence has been labeled by a unique number was given in Figures 2 and 3 (named X) followed by the sampling ID labeling. [file 1471-2180-14-110-S5.docx]

**Supplementary Table 4:** Description of the V4 sequences ID labeling. Each sequence has been labeled by a unique number was given in Figure 1 and 2A (named X in the table below) followed by the sampling ID labeling.

| Sequence sampling-ID | Location | Year | Template | Size fraction | Depth | Number of sequences |
| --- | --- | --- | --- | --- | --- | --- |
| X-0005 | Naples | 2009 | cDNA | [total] | Sediment | 45 |
| X-0006 | Oslo | 2009 | cDNA | [total] | Sediment | 2 |
| X-0007 | Oslo | 2009 | DNA | [total] | Sediment | 6 |
| X-0008 | Naples | 2009 | DNA | [total] | Sediment | 64 |
| X-0009 | Oslo | 2009 | DNA | [total] | Sediment | 3 |
| X-0010 | Oslo | 2009 | cDNA | [total] | Sediment | 3 |
| X-0042 | Naples | 2009 | DNA | [0.8-3] | DCM | 1 |
| X-0068 | Barcelona | 2010 | cDNA | [total] | Sediment | 1 |
| X-0072 | Roscoff | 2010 | cDNA | [total] | Sediment | 2 |
| X-0078 | Barcelona | 2010 | DNA | [total] | Sediment | 1 |
| X-0081 | Roscoff | 2010 | DNA | [0.8-3] | Subsurface | 3 |
| X-0082 | Roscoff | 2010 | DNA | [total] | Sediment | 8 |
| X-0089 | Naples | 2010 | cDNA | [total] | Sediment | 3 |
| X-0099 | Naples | 2010 | DNA | [total] | Sediment | 24 |
| X-0108 | Oslo | 2010 | cDNA | [20-total] | Subsurface | 4 |
| X-0116 | Oslo | 2010 | DNA | [0.8-3] | Subsurface | 3 |
| X-0118 | Oslo | 2010 | DNA | [20-total] | Subsurface | 3 |
| X-0130 | Oslo | 2009 | cDNA | [total] | Sediment | 2 |
| X-0134 | Oslo | 2010 | cDNA | [total] | Sediment | 1 |
| X-0136 | Oslo | 2009 | DNA | [3-20] | DCM | 1 |
| X-0137 | Oslo | 2009 | DNA | [0.8-3] | DCM | 1 |
| X-0139 | Oslo | 2009 | DNA | [total] | Sediment | 2 |
| X-0140 | Oslo | 2009 | DNA | [total] | Sediment | 39 |
| X-0141 | Oslo | 2009 | DNA | [0.8-3] | Subsurface | 3 |
| S0143 | Oslo | 2010 | DNA | [0.8-3] | DCM | 1 |
| S0144 | Oslo | 2010 | DNA | [total] | Sediment | 9 |
| S0147 | Naples | 2009 | cDNA | [total] | Sediment | 9 |
| S0148 | Naples | 2009 | DNA | [total] | Sediment | 20 |
| S0168 | Naples | 2009 | DNA | [0.8-3] | DCM | 1 |
