# Supplementary material for: Diverse molecular signatures for ribosomally ‘active’ Perkinsea in marine sediments
Source: BMC Microbiol. 2014 Apr 29;14:110. doi: 10.1186/1471-2180-14-110 (PMC4044210; doi:10.1186/1471-2180-14-110)
Supplement: Additional file 6: Table S5 — Bayesian model comparison for method selection in phylogenetic inference. [file 1471-2180-14-110-S6.doc]

**Supplementary Table 5:** Bayesian model comparison for method selection in phylogenetic inference.

|  | Model_Covarion_ / Model_Non-covarion_ (*Log_10_ Bayes Factors*) |
| --- | --- |
| Full-length analysis | 730.12 |
| V4 region analysis | 254.34 |
